# Felis catus papillomavirus type-2 E6 binds to E6AP, promotes E6AP/p53 binding and enhances p53 proteasomal degradation

**DOI:** 10.1038/s41598-018-35723-7

**Published:** 2018-12-03

**Authors:** Gennaro Altamura, Karen Power, Manuela Martano, Barbara degli Uberti, Giorgio Galiero, Giovanna De Luca, Paola Maiolino, Giuseppe Borzacchiello

**Affiliations:** 1 Department of Veterinary medicine and Animal productions – University of Naples Federico II – Via Veterinaria 1, Naples, 80137 Italy; 2 Istituto Zooprofilattico Sperimentale del Mezzogiorno, Via Salute 2, Portici, Naples, 80055 Italy

## Abstract

E6 from high risk human papillomaviruses (HR HPVs) promotes ubiquitination and degradation of p53 tumour suppressor by mediating its binding to ubiquitin ligase E6AP in a ternary complex, contributing to cell transformation in cervical cancer. We have previously shown that *Felis catus* papillomavirus type −2 (FcaPV-2) E6 is expressed in feline squamous cell carcinoma (SCC) and displays the ability to bind p53 and decrease its protein levels in transfected CRFK cells. However, the mechanism of p53 downregulation has not yet been characterized. Here we show that FcaPV-2 E6 bound to E6AP, which in turn was bound by p53 exclusively in cells expressing the viral oncoprotein (CRFKE6). Furthermore, p53 was highly poly-ubiquitinated and underwent accumulation upon E6AP gene knockdown in CRFKE6. Half-life experiments and proteasome inhibition treatments indicated that down-regulation of p53 protein in CRFKE6 was due to accelerated proteasomal degradation. E6AP/p53 binding was also demonstrated in two feline SCC cell lines expressing FcaPV-2 E6, where p53 protein levels and poly-ubiquitination degree were proportional to E6 mRNA levels. The data obtained in both artificial and spontaneous *in vitro* models suggest that FcaPV-2 E6 degrades p53 through a molecular mechanism similar to HR HPVs, possibly contributing to the development of feline SCC.

## Introduction

Papillomaviruses (PVs) are oncogenic DNA viruses that induce neoplastic lesions of skin and mucosal epithelia in humans and animal species, including the domestic cat (*Felis catus*)^1^. The transforming potential of human and some animal PVs is mostly due to the biological activity of E6 and E7 oncogenes, whose main function is to force the infected cells to re-enter the cell cycle, in order to exploit the host cell duplication machinery in favour of viral genome replication. In this context, E6 and E7 hijack the cell fate towards impaired proliferation and promote neoplastic transformation^1^. The major cellular target of E6 oncoproteins is the tumour suppressor p53, a transcription factor which positively regulates several genes involved in cell cycle arrest and/or apoptosis upon different types of cell stress^2^.

Alpha high-risk (HR) human PV types-16/-18 (HPV-16/-18) are clearly associated with cervical cancer and the mechanism by which their E6 down-regulate p53 has been extensively investigated and well characterized. It involves the assembly of a ternary complex of E6 with p53 and the cellular ubiquitin ligase E6AP in a precise temporal sequence of molecular events: once E6 is bound to E6AP, the heterodimer E6/E6AP recruits p53 and causes its enhanced ubiquitination and accelerated proteasomal degradation, leading to the loss of tumour suppressor functions and therefore contributing to neoplastic process^2,3^. It is widely recognized that HR HPVs, particularly HPV-16, are also involved in the pathogenesis of a sub-group of oropharyngeal squamous cell carcinoma (SCC) by exerting the same transforming mechanisms^4^. This type of oral cancer forms a distinct category of head and neck SCC (HNSCC), with different markers and prognosis with respect to that associated with other risk factors such as tobacco smoke and alcohol consumption^4^.

In cats, *Felis catus* PV type −2 (FcaPV-2) is an emerging oncogenic virus: it is highly associated with feline SCC and its pre-neoplastic precursors, where expression of viral oncogenes has been widely reported^1,5^. Notably, feline oral SCC is considered a spontaneous animal model of human HNSCC^6,7^. However, its association with FcaPV-2 is infrequent so far; thus whether PVs infection may represent a possible risk factor as in human counterpart is still unclear^5,8–12^. FcaPV-2 E6 and E7 open reading frames (ORFs) have been cloned and the transforming properties of the resulting oncoproteins in part elucidated^5,13^. Particularly, it has been demonstrated that FcaPV-2 E6 is able to bind p53 and its ectopic expression in feline cells results in decreased p53 protein levels, suggesting biological similarities to the E6 from HR HPV types^5^. However, the molecular mechanism of p53 down-regulation by FcaPV-2 E6 has not yet been characterized.

The aim of this study was to unravel the mechanism of p53 down-regulation by FcaPV-2 E6 in living cells and whether it involves E6AP and proteasome pathway in our functional model of FcaPV-2 driven pathogenesis. Additionally, similar molecular studies were extended to cell lines derived from spontaneous feline oral SCC, in order to describe their molecular scenario and hypothesize whether it might take place also in an *in vitro* model of naturally occurring cancer.

## Methods

### Cell culture and treatments

Crandel-Rees feline kidney (CRFK) cells stably expressing empty pCEFL-HA (CRFKpCEFL) or FcaPV-2 E6 tagged with HA epitope (CRFKE6) have been generated in our laboratory and cultured as previously reported^5^. Cervical carcinoma Hela cells harbouring HPV-18 were purchased at ATCC cell bank. Feline oral squamous cell carcinoma cell lines SCCF2 and SCCF3 developed in the Rosol laboratory are a kind gift from Professor T.J. Rosol (The Ohio State University) and have been cultured as described elsewhere^14–16^.

For p53 half-life experiments, 2 × 10^5^ cells were seeded in 6-well plates and, after 24 hours (h), treated with the protein synthesis inhibitor cycloheximide (Sigma #C7698-1G) at 20 µg/mL for 0, 0.5, 1, 2.5, 5 h, or with the proteasome inhibitor MG132 (Sigma #C2211-5MG) at 30 µM for 4 h. In control plates, drugs were replaced with sterile water or DMSO. Treated cells were harvested and analysed by WB as described below.

### Western blotting

Total protein extraction, protein quantification, sodium dodecyl sulfate (SDS)-polyacrylamide gel electrophoresis (PAGE) and Western blotting (WB) were performed as described previously^13^. Primary antibodies to the following proteins were applied overnight (O/N) at 4 °C at 1:1000 dilution: p53 (Santa Cruz Biotechnology #sc-6243), E6AP (Sigma #E8655), HA (Santa Cruz Biotechnology #sc-7392), ubiquitin (Santa Cruz Biotechnology #sc-8017) and β-actin (Calbiochem #CP01-1EA). Protein band detection and densitometric analysis were performed as reported elsewhere^13^. Protein expression levels were normalized to β-actin.

### Co-immunoprecipitation

Total protein lysates were obtained as described above from three 100 mm Petri dishes at 100% confluence. For each cell type, 2 mg of proteins were pre-cleared by incubation with 30 µL of A-G/plus sepharose beads (Santa Cruz Biotechnology #sc-2003) for 1 h at 4 °C with gentle agitation. An aliquot of each sample was kept before immunoprecipitation as input. Protein lysates were incubated O/N at 4 °C on a rocking wheel, with anti-p53 antibody (2 μg/mL, 1:100), anti-E6AP antibody (20 μg/mL, 1:100), anti-HA antibody (Santa Cruz Biotechnology #sc-805, 8 μg/mL, 1:25) or non-specific rabbit IgG (Bethyl Laboratories, Inc, #P120-201) at the same concentrations as control. Then, 30 µL of beads were added to the samples, and the mixture was rotated for 2 h at 4 °C. After 3 washing steps in lysis buffer, the immunoprecipitates were resuspended in Laemmli sample buffer and analysed by WB for p53, E6AP, HA and ubiquitin. Appropriate blocking reagents (Genetex #425858) and HRP conjugated anti-rabbit secondary antibodies with no affinity for denatured IgG (Genetex #221666-01) were used to mask IgG bands and avoid overlapping with specific protein bands. The fraction of p53 bound by E6AP was estimated by densitometric analysis of immunoprecipitation (IP) and input bands at the same exposure times, taking into consideration the percentage of total loaded in input lane.

### Double-immunofluorescence staining

Cells grown for 2 days on coverslips were washed, fixed, permeabilized and subjected to background blocking as previously reported^17^. Anti-HA, anti-p53 and anti-E6AP antibodies were applied for 2 h at room temperature (rt) in a humidified chamber at 1:50 dilution in PBS. Then, slides were washed three times with PBS and incubated with Alexa Fluor 488 goat anti-rabbit (Thermo Fisher Scientific #A11008) and Alexa Fluor 546 goat anti-mouse (Thermo Fisher Scientific #A11030) for 30 min at rt in a humidified chamber at 1:100 dilution. Finally, after washing with PBS, the slides were mounted in aqueous medium PBS:Glycerol 1:1 containing DAPI (1:1000) to allow nuclear counter-staining. For scanning and photography, slides were read under ZOE Fluorescent Cell Imager (Bio-Rad Laboratories).

### Gene silencing

E6AP gene silencing was achieved by using antisense oligonucleotides pool ON-TARGETplus Human UBE3A (7337) siRNA - SMARTpool (Dharmacon #L-005137-00-0005) at 30 nM (CRFK) or 10 nM (Hela). Three custom synthetized siRNA oligonucleotides were synthetized to knock down FcaPV-2 E6 gene expression (Silencer@ Select, Ambion #4399666) based on the sequence of viral gene (Genbank: EU796884.1). The sequences of the oligonucleotides were as follows: (1) Sense: 5′-GUAUUUUGCGGAACACUUAtt-3′, Antisense: 5′-UAAGUGUUCCGCAAAAUACgc-3′; (2) Sense: 5′-GGACUUUGCAUGAGUGGUAtt-3′, Antisene: 5′-UACCACUCAUGCAAAGUCCag-3′; (3) Sense:5′-AGACCAUUUUGAUUAUGCAtt-3′, Antisense: 5′-UGCAUAAUCAAAUGGUCUag-3′. A pool of the three oligonucleotides was employed at 50 nM. Cells were plated at 2 × 10^5^ density in 6-well plates and, after 24 h, siRNA or scrambled RNA (Ambion #4390843) at same concentrations were transfected by using Lipofectamine 2000 (Thermo Fisher Scientific #11668027) according to the standard protocol. Cells were harvested after 48 h and analysed by WB and Real-time PCR.

### DNA extraction and Real-time PCR for detection of viral DNA

DNA from formalin-fixed paraffin embedded (FFPE) tissues and cultured cells was extracted by using the DNA Blood and Tissue kit (Qiagen #69504) following the manufacturer recommendations. Real-time PCR for amplification of a specific fragment of FcaPV-2 E6 from SCCF2 and SCCF3 cell lines was performed by using iTaq Universal SYBR Green Supermix (Bio-Rad Laboratories #1725121) according to the brand instructions, by using the primers described elsewhere^18^. DNA from CRFKpCEFL and CRFKE6 were run concomitantly as negative and positive controls, respectively. FcaPV-2 cloned genome and one sample with no template were analysed as further controls. Melting curve analysis was performed and the melting peak compared with that obtained in the positive control to confirm the specificity of the amplicon. Amplification products were submitted to sequence analysis (BMR-Genomics) and the obtained sequences aligned to the viral gene sequence using the Basic Local Alignment Search Tool (NCBI/BLAST). In tissue samples, Real- time PCR for detection of viral DNA was performed as previously described^19^. Feline β-globin was amplified as previously reported to ensure the presence of amplifiable DNA in each cell and tissue sample^18^.

### RNA extraction, reverse transcription (RT) and Real-time quantitative PCR (qPCR) for detection of FcaPV-2 mRNA

Total RNA was extracted from cells using the RNeasy Mini Kit (Qiagen #73504) following the manufacturer recommendations and subjected to DNAse digestion (Roche #04536282001). RT was performed on 1 µg of RNA using iScript cDNA Synthesis Kit (Bio-Rad Laboratories #1708890); RT without the addition of reverse transcriptase enzyme was also performed on each RNA sample as control. For each sample, 50 ng of cDNA were subjected to Real-time qPCR as described above to amplify a segment of FcaPV-2 E6 and p53 transcript employing the primers detailed elsewhere^18^. Amplification of feline β-2-microglobulin (β2MG) was performed in parallel to allow normalization of the results as previously reported^20^. Bio-Rad CFX Manager software was used to generate gene expression data based on the 2^−ΔΔCT^ method.

RNA was obtained from paraffin embedded tissues by using RNeasy FFPE Kit (Qiagen #73504) and retro-transcribed as above. RT-PCR for amplification of a 111 bp fragment expected to be in the transcript encoding for E6 and E7 proteins was performed as previously described^19^.

### Tissue samples

Seventeen FFPE feline SCC (T1-T17) were retrieved from the archive of the “Istituto Zooprofilattico Sperimentale del Mezzogiorno” and Department of Veterinary medicine and Animal productions – University of Naples Federico II. Details on animals’ breed, age, anatomical sites of sampling and diagnosis are summarized in Table 1. For each tissue sample, 5 µm thick sections were cut with separate microtome blades and stored in sterile tubes for molecular analysis or onto slides for immuno-staining.

**Table 1:** FcaPV-2 DNA detection and mRNA expression in feline SCC samples (ESH: European short haired; UK: unknown; SCC: squamous cell carcinoma).

Sample	Breed	Age (years)	Anatomical site	Diagnosis	FcaPV-2 DNA	FcaPV-2 mRNA
T1	ESH	6	Face (Chin)	SCC	+	+
T2	ESH	15	UK	SCC	+	+
T3	UK	6	Right ear	SCC	+	+
T4	UK	UK	Nasal planum	SCC	+	+
T5	ESH	12	Mandible	SCC	+	+
T6	ESH	10	Right ear	SCC	−	−
T7	ESH	6	Left ear	SCC	−	−
T8	UK	6	Lower right eyelid	SCC	−	−
T9	ESH	15	Right ear	SCC	−	−
T10	ESH	4	Ears	SCC	−	−
T11	UK	UK	UK	SCC	−	−
T12	ESH	2	UK	SCC	−	−
T13	ESH	14	UK	SCC	−	−
T14	ESH	12	UK	SCC	−	−
T15	ESH	15	UK	SCC	−	−
T16	ESH	10	UK	SCC	−	−
T17	ESH	10	UK	SCC	−	−

### Immunohistochemistry (IHC)

To assess the possible co-expression of p53 and E6AP in the same cells within the SCC, serial sections were immunostained using streptavidin-avidin method (Mach 1 Universal HRP-Polymer Detection, Biocare Medical #M1U539 G, L10). They were mounted on amino-silane-coated slides, dewaxed in xylene and rehydrated through graded alcohols. Slides were incubated in hydrogen peroxide 0.3% and absolute methanol solution (4:1) for 20 min to quench endogenous peroxidase. Antigen retrieval was carried out by two cycles of microwave-treatment, at 500 W for 5 min and 700 W for 5 min, in 0.01 M citrate buffer (pH 6.0). Then slides were washed in 0.01 M phosphate-buffered saline (PBS; pH 7.2–7.4) and incubated in Background Sniper solution for 30 min to block non-specific bindings. Primary antibodies at 1:50 dilution were applied overnight at 4 °C. Then, sections were rinsed twice for 5 min in PBS and incubated for 20 min at rt with appropriate biotinylated secondary antibodies. Following a rinsing step in PBS, streptavidin-conjugated horseradish peroxidase was applied for 30 min at rt. The reaction was developed with 3,3′-diaminobenzidine terahydrochloride and sections were counterstained with Mayer’s haematoxylin.

The same reaction was run on slides with primary antibodies omitted as negative control, feline mammary adenocarcinoma was used as positive control for p53 antibody^21^.

### Statistical analysis

For statistical analysis, Student’s t-test was performed using SPSS 17.0 software (SPSS Inc., Chicago, ILL, USA) and differences considered statistically significant for P < 0.05 (*) or 0.01 (**).

## Results

### Expression and subcellular localization of FcaPV-2 E6, p53 and E6AP in feline epithelial cells expressing FcaPV-2 E6

**Figure 1:**
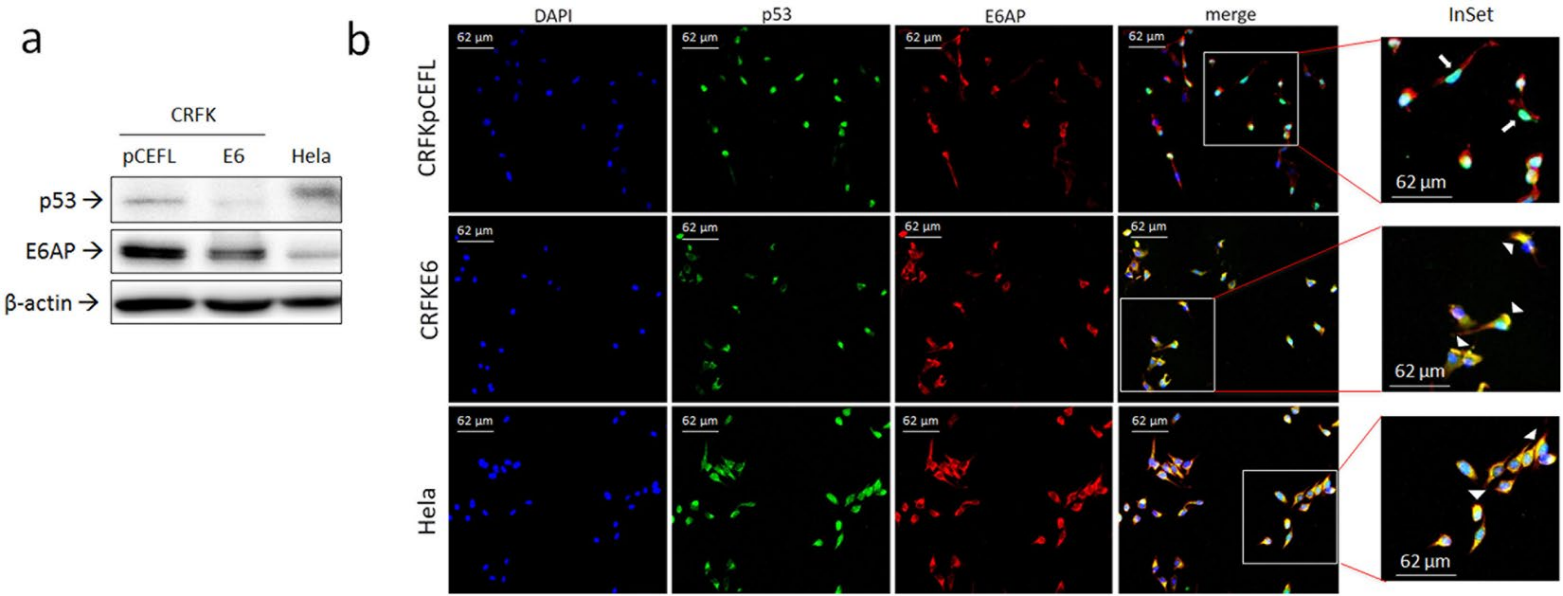
Expression and localization of p53 and E6AP in CRFK cells expressing FcaPV-2 E6. (**a**) CRFK cells stably transfected with FcaPV-2 E6 (CRFKE6) and empty pCEFL-HA (CRFKpCEFL) were lysed and analysed by WB by using anti-p53 and anti-E6AP specific antibodies. Hela whole cell lysate was run along with feline samples to ensure the specificity of the protein bands. The membrane was stripped and reprobed with anti-β-actin antibody to ensure equal protein loading in each lane. A representative WB out of three independent experiments, demonstrating down-regulation of p53 and E6AP in cells expressing FcaPV-2 E6, is shown. (**b**) CRFKpCEFL and CRFKE6 were grown on coverslips and subjected to double indirect IF staining for p53 (green fluorescence) and E6AP (red fluorescence). Nuclei were counterstained with DAPI. Slides were read under ZOE™ Fluorescent Cell Imager (Bio-Rad Laboratories). InSet shows higher magnification of merge panel. Representative images showing nuclear localization of p53 in CRKpCEFL (white arrow) and perinuclear-like co-localization of p53 and E6AP in CRFKE6 and Hela (yellow fluorescence, white arrowheads) are shown.

Whole cell lysates from transfected CRFK cells were first analysed by WB followed by densitometric analysis for p53 and E6AP (Fig. 1, Supplemental Fig. S1). Lower p53 protein levels were confirmed in CRFKE6 compared to CRFKpCEFL (Fig. 1a, Supplemental Fig. S1a), as already reported^5^.

Interestingly, E6AP protein was expressed and down-regulated in CRFKE6 with respect to CRFKpCEFL (Fig. 1a, Supplemental Fig. S1b). Hela whole cell lysate run along with feline cells as antibody control confirmed the identity of the bands.

To investigate the intracellular localization of p53 and E6AP proteins and possibly hypothesize the involvement of E6AP in p53 impairment, cells were subjected to double immunofluorescence (IF) staining (Fig. 1b). In CRFKpCEFL, p53 (green staining) was mainly localized in the nuclei, as judged by the merge with DAPI blue fluorescence, whilst E6AP protein (red staining) was detected in the cytoplasm of cells. Differently, p53 was expressed mostly in the cytoplasm of CRFKE6, where it markedly co-localized with E6AP in a perinuclear like region, as indicated by the yellow fluorescence due to the merge of green and red staining.

In accordance with previous studies, p53 expression in Hela cells was detected at cytoplasmic/perinuclear level^22^, where it co-localized with E6AP.

Furthermore, CRFKE6 cells were stained for HA (red fluorescence), to detect HA-tagged FcaPV-2 E6, and p53 (green fluorescence) by double IF (Supplemental Fig. S2). FcaPV-2 E6 was detected in the cytoplasm of cells resembling what previously reported for HR-HPVs E6^22^, and it co-localized with p53 in the cytoplasm, in a perinuclear like position, as pointed out by yellow fluorescence.

### Interaction of E6AP with FcaPV-2 E6 and p53

**Figure 2:**
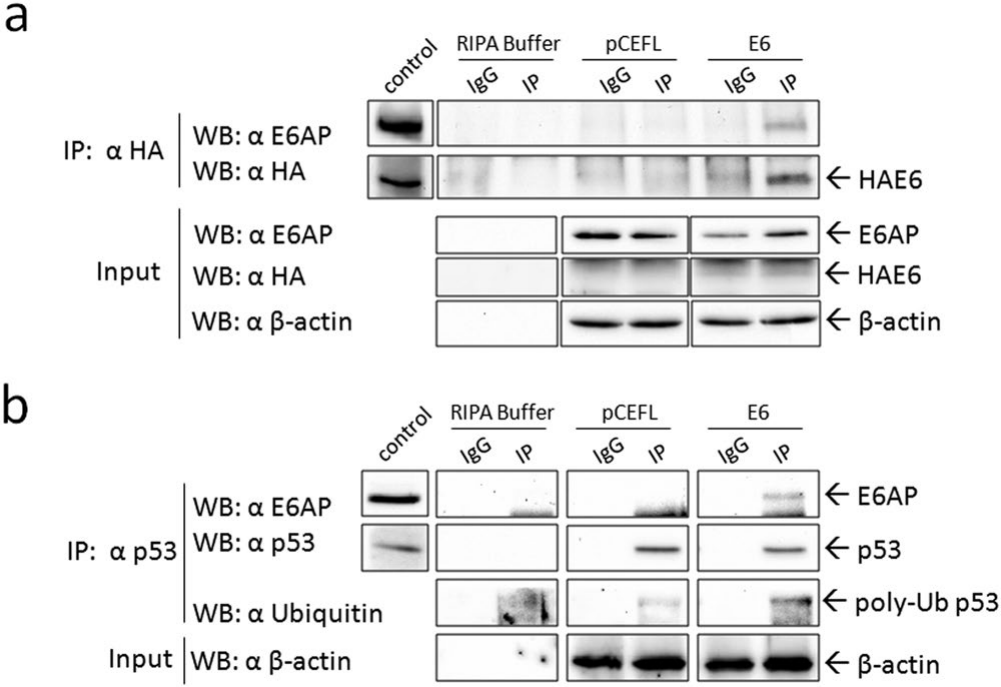
Co-immunoprecipitation (co-IP) of E6AP with FcaPV-2 E6 and p53 in CRFKE6. (**a**) Cell lysates from CRFKE6 and CRFKpCEFL were incubated with anti-HA antibody for immunoprecipitation (IP) of HA-tagged FcaPV-2 E6 or with non-specific antibody (IgG) as control. E6AP was revealed by WB upon incubation with the specific antibody. The membrane was stripped and blotted with anti-HA antibody to confirm the occurrence of the IP. CRFKE6 cell lysate was loaded to check for molecular weight of the IP bands (control). An aliquot of samples was kept before IP as input and analysed by WB for E6AP, HAE6 and for β-actin to ensure that equal amount of proteins were used for each cell line. Boxes of IP are cut from the same gel at the same exposure times, control square is cut at a different exposure time and properly aligned according to the molecular weight standard loaded onto the gel. Full length blots with molecular markers are shown in Supplementary information (Supplemental Fig. S11). A representative co-IP out of two independent experiments, demonstrating physical interaction of E6AP and FcaPV-2 E6, is shown. (**b**) p53 was immunoprecipitated by incubation of cell lysates with specific antibody and the presence of E6AP and ubiquitin in the immunocomplex revealed by WB. The same membrane was blotted for p53 to confirm the IP. The molecular weight of IP bands was checked by loading whole cell lysate along with IP samples. Input were analysed for β-actin as above. A representative co-IP out of two independent experiments, demonstrating interaction of E6AP with p53 and high poly-ubiquitination of p53 in CRFKE6, is shown.

Co-localization of p53 and E6AP in cells expressing E6 prompted us to investigate their possible physical interaction by co-immunoprecipitation (co-IP) assays. In the HPV pathogenic model, the assembly of E6/E6AP heterodimer is essential for the recruitment of p53 in the molecular complex^3^. Therefore, we first verified whether FcaPV-2 E6 was able to recruit E6AP in our feline cell system (Fig. 2a). The viral oncoprotein was successfully immunoprecipitated by using anti-HA antibody and, importantly, E6AP was pulled-down exclusively in the HAE6 IP lane, suggesting their molecular interaction (Fig. 2a).

Then, interaction of E6AP with p53 was investigated (Fig. 2b). Lower p53 amounts were immunoprecipitated by using a specific antibody in CRFKE6 compared to CRFKpCEFL as confirmed by densitometric analysis (Supplemental Fig. S3a), consistently with WB results. Notably, E6AP protein was co-immunoprecipitated with p53 exclusively in CRFKE6 (Fig. 2b), consistently with co-localization data. As expected, the same procedure performed on Hela cells as positive control, yielded the co-IP of E6AP with p53 (not shown). The p53 and E6AP bands detected in CRFKE6 whole cell lysates loaded as control, confirmed the identity of co-IP bands. Comparable β-actin levels revealed in input samples confirmed that equal amounts of proteins were immunoprecipitated in each cell sample.

The physical interaction between the ubiquitin ligase E6AP and p53 in the ternary complex is known to enhance poly-ubiquitination of p53^2^. Thus, IP samples were analysed by WB with an anti-ubiquitin antibody: a high molecular weight band, likely corresponding to poly-ubiquitinated p53^23–25^, was detected in CRFKE6 IP lane, at higher levels compared to CRFKpCEFL (Fig. 2b and Supplemental Fig. S3b). Consistently, E6 gene knock-down by siRNA induced p53 rescue along with a change in ubiquitination pattern (Supplemental Fig. S4a). The ratio between poly-ubiquitinated p53 and total p53 was decreased in E6 knocked-down with respect to scramble treated cells (Supplemental Fig. S4b), further suggesting that FcaPV-2 E6 is responsible for p53 ubiquitination and subsequent degradation.

### Analysis of p53 half-life and proteasomal degradation

**Figure 3:**
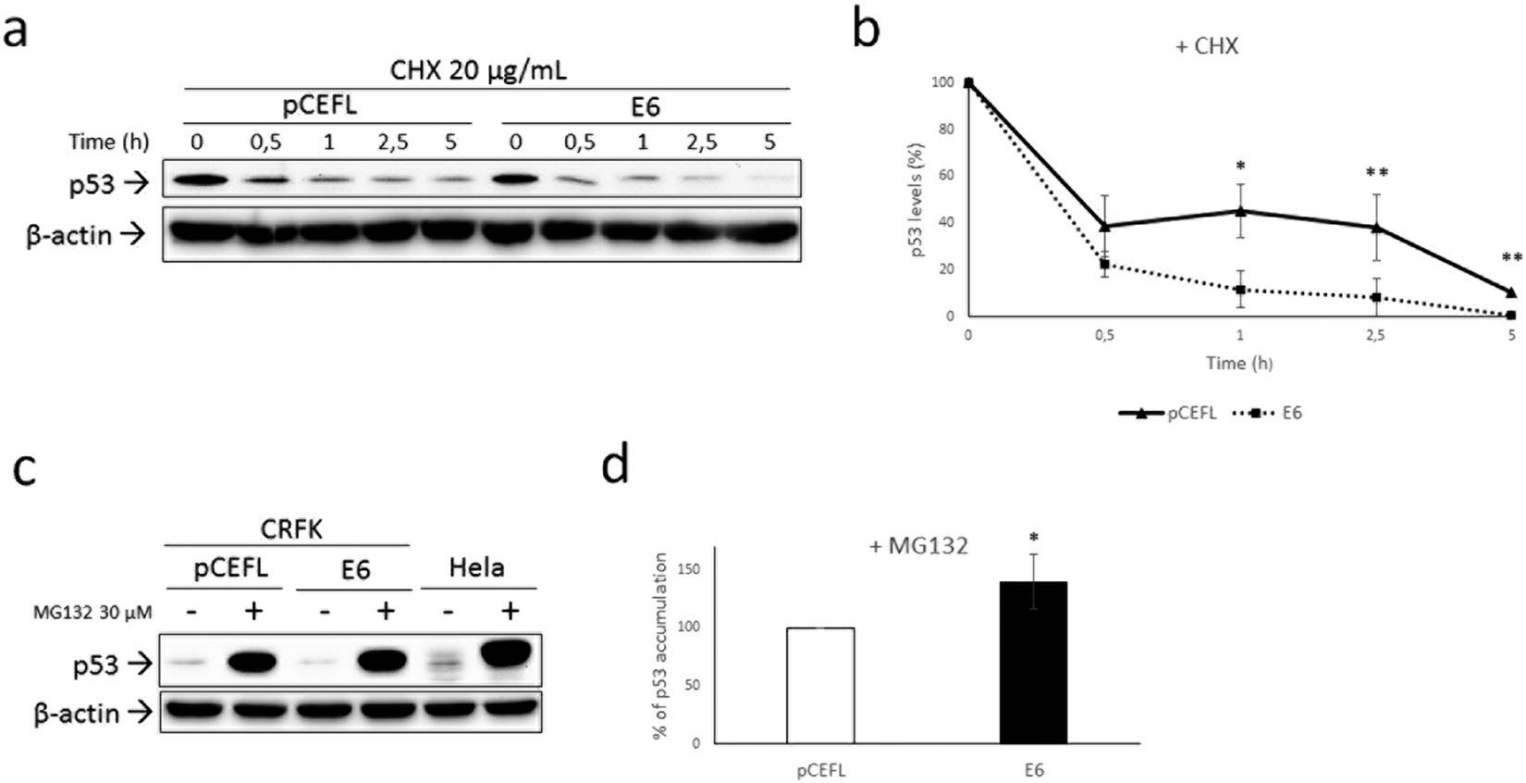
Analysis of p53 half-life and proteasomal degradation. (**a**) CRFKpCEFL and CRFKE6 were treated with protein synthesis inhibitor cycloheximide (20 µg/mL) and collected at different time points indicated in hours (h). Cells were lysed and analysed by WB for p53, then the blot was stripped and reprobed for β-actin to ensure equal protein loading in each lane and allow normalization. A representative gel demonstrating p53 shorter half-life in presence of FcaPV-2 E6 is shown. (**b**) The graph shows the quantification of p53 bands, normalized to β-actin levels and calibrated against the levels of p53 at time 0 of treatment (set as 100%). Results shown are the means +/− standard deviation (SD) of at least three independent experiments and demonstrate accelerated p53 degradation in CRFKE6 compared to CRFKpCEFL at each time point (Time 1: P = 0.057; Time 2.5: P = 0.0345; Time 5: P = 0,0034). (**c**) CRFKpCEFL and CRFKE6 were treated with the proteasome inhibitor MG132 at 30 µM for 4 h and collected to be analysed by WB for p53. Hela were concomitantly analysed as control. The blot was stripped and reprobed for β-actin to ensure equal protein loading in each lane and allow normalization. (**d**) Accumulation upon MG132 treatment in CRFKpCEFL with respect to untreated cells was calculated by densitometric quantification of p53 bands normalized to β-actin levels and arbitrarily set as 100% (white bar). The same calculation was applied to treated *vs* untreated CRFKE6 and the difference in the % of accumulation obtained by ratio with the value pointed out in CRFKpCEFL (black bar). Results shown are the means +/− SD of four independent experiments and demonstrate higher % of p53 accumulation in CRFKE6 compared to CRFKpCEFL (P = 0.0152).

E6-driven, E6AP dependent poly-ubiquitination of p53 leads to its accelerated proteasomal degradation^2^. Hence, we analysed the half-life of p53 protein upon treatment with cycloheximide at different time points. The results from repeated, independent experiments indicated that p53 half-life was shorter in CRFKE6 compared to CRFKpCEFL (Fig. 3a). Most importantly, the percentage of protein decrease at each time point with respect to time 0 was higher in cells expressing the oncoprotein, suggesting accelerated p53 degradation (Fig. 3b). To further investigate the nature of this finding, cells were treated with proteasome inhibitor MG132 and analysed by WB. p53 destabilization in CRFKE6 compared to CRFKpCEFL was confirmed in untreated cells (Supplemental Fig. S5). As expected, p53 accumulation was revealed in both cell lines with respect to the untreated counterpart (Fig. 3c and Supplemental Fig. S5), since p53 half-life is physiologically regulated by the proteasome^26^. However, if considering the protein increase in CRFKpCEFL as 100%, a mean ∼40% higher p53 accumulation was obtained in CRFKE6 (Fig. 3d), suggesting that the enhanced p53 degradation was proteasomal dependent.

### Analysis of p53 rescue upon E6AP gene silencing

**Figure 4:**
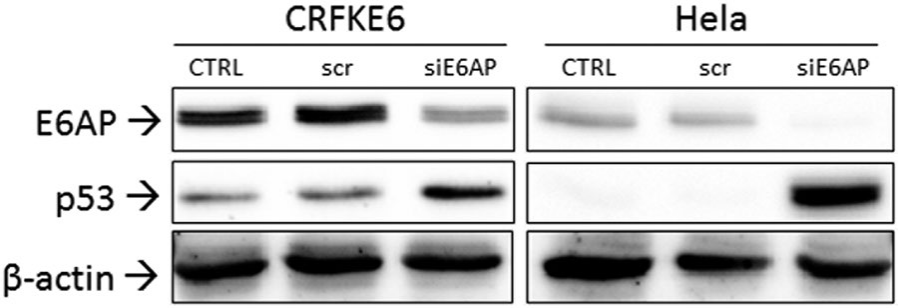
Analysis of p53 rescue upon E6AP gene silencing. CRFKE6 were transiently transfected with 30 nM siRNA directed against E6AP (siE6AP) or with scramble RNA (scr) and analysed by WB for p53 and E6AP. Hela were subjected to the same procedure as control. The blot was stripped and reprobed for β-actin to ensure that equal amounts of protein were loaded in each lane. A representative gel out of two independent experiments, shows accumulation of p53 in correspondence of inhibition of E6AP expression (CTRL: untreated control cells).

To further analyse whether p53 degradation was E6AP dependent in FcaPV-2 E6 transfected cells, E6AP gene expression was knocked down by siRNA procedures and cells were analysed by WB followed by densitometric analysis to evaluate the rescue of p53. The results revealed that p53 accumulated upon E6AP gene silencing in CRFKE6 compared to scramble-treated and untreated cells, as well as in Hela cells analysed in parallel as positive control (Fig. 4 and Supplemental Fig. S6). Conversely, the same procedure applied to CRFKpCEFL did not yield p53 accumulation (Supplemental Fig. S7).

### Detection of FcaPV-2 DNA and E6 gene expression in feline oral SCC cell lines

**Figure 5:**
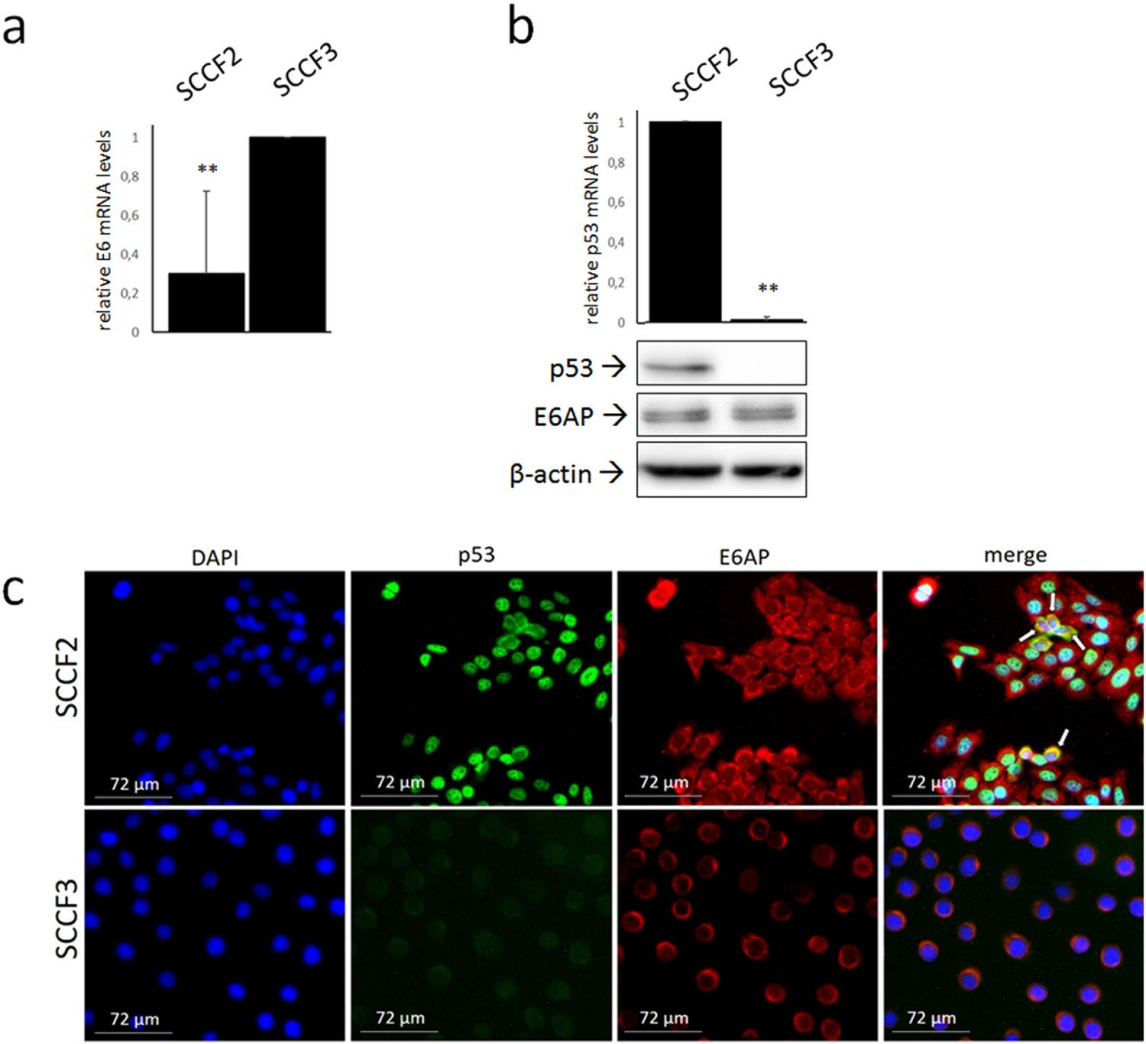
FcaPV-2 E6 mRNA levels, expression and localization of p53 and E6AP in feline oral SCC cell lines. (**a**) SCCF2 and SCCF3 cells were subjected to RNA extraction, reverse transcription and Real time quantitative PCR (qPCR) to analyse FcaPV-2 E6 gene expression. Results were normalised for β2-microglobulin expression. The graph shows the results as mean +/− standard deviations (SD) of five independent experiments where the cell line with higher E6 expression levels (SCCF3) (P = 0.0062) was arbitrarily set as calibrator. (**b**) Cells were analysed by Real-time qPCR for p53 gene expression and results normalized as above. The graph represents mean +/− SD from three independent experiment and demonstrates p53 down-regulation in SCCF3 compared to SCCF2 set as calibrator (P = 0.00). Representative WB gels for p53 and E6AP are also shown. Stripping and reprobing with β-actin confirmed equal protein loadings. (**c**) SCCF2 and SCCF3 cells were grown on coverslips and subjected to double IF staining for p53 (green fluorescence) and E6AP (red fluorescence), nuclei were counterstained with DAPI. Representative fields showing nuclear or cytoplasmic/perinuclear p53 expression in SCCF2 and undetectable p53 staining in SCCF3 are illustrated. White arrows indicate perinuclear co-localization (yellow fluorescence) of the two proteins in a sub-set of SCCF2 cells in the merge panel.

To strengthen the previous results in an *in vitro* model derived from naturally occurring cancer, feline oral SCC cell lines SCCF2 and SCCF3 were subjected to an experimental workflow similar to the previous cell model. First, cells were analysed by qualitative Real-time PCR for detection of a FcaPV-2 DNA sequence localized within the E6 gene. Successful amplification was obtained in SCCF2, SCCF3 and, as expected, in CRFKE6 but not CRFKpCEFL run in parallel as positive and negative control, respectively (data not shown). FcaPV-2 genome was amplified as further positive control, one sample with no DNA did not yield amplification. Sequence analysis confirmed the identity of the amplicons. Then, RT and Real-time qPCR were performed to verify whether FcaPV-2 E6 was expressed in these cell lines: the results from repeated, independent experiments yielded higher E6 expression in SCCF3 compared to SCCF2 (Fig. 5a).

### Expression and localization of p53 and E6AP in feline oral SCC cell lines

Then, to investigate whether FcaPV-2 E6 might influence p53 and E6AP status in these cell lines, cells were analysed by WB and double IF staining. The p53 band was readily detected in SCCF2 cells but not in SCCF3 cell line (Fig. 5b), where IP or enhanced ECL reagent and high exposure time could yield a faint band (see below). To further investigate this finding, p53 gene expression was analysed by Real-time qPCR. Lower p53 mRNA levels were revealed in SCCF3 (Fig. 5b), explaining, at least in part, WB results. E6AP protein expression was detected in SCCF2 and SCCF3 cell lines (Fig. 5b).

In SCCF2 cells, p53 staining (green fluorescence) was nuclear in most of the cells as it merged with DAPI blue staining, however it also co-localized in a cytoplasmic, juxta-nuclear position with E6AP (red fluorescence) in a sub-set of cells per field as judged by yellow fluorescence (Fig. 5c). E6AP expression was mostly perinuclear in SCCF3, whilst green p53 staining was undetectable (Fig. 5c), consistently with WB results.

### E6AP binds to p53 in FcaPV-2 positive feline oral SCC cell lines

**Figure 6:**
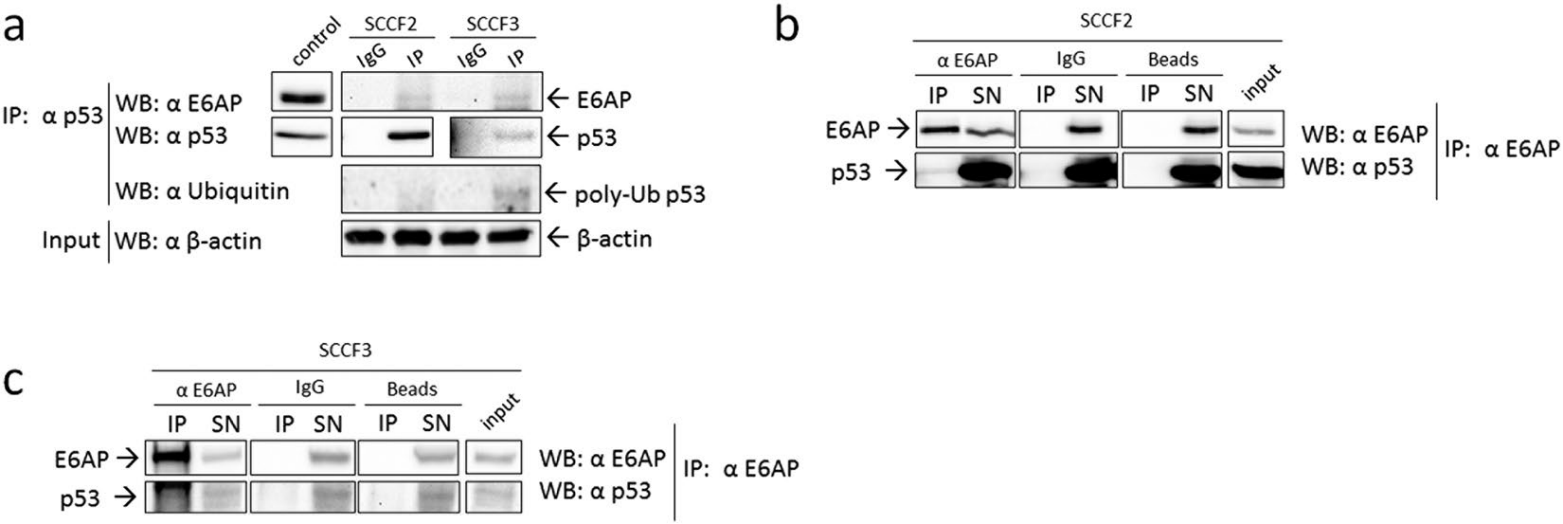
E6AP/p53 physical interaction in feline oral SCC cells expressing FcaPV-2 E6. (**a**) Cell lysates from SCCF2 and SCCF3 were incubated with anti-p53 antibody for immunoprecipitation (IP) or with non-specific antibody (IgG) as control. E6AP and ubiquitin were revealed by WB upon incubation of the membrane with the specific antibodies. The membrane was stripped and blotted with anti-p53 antibody to confirm the occurrence of the IP. SCCF2 cell lysate was loaded to check for molecular weight of the IP bands (control). An aliquot of IP samples was kept before IP as input and analysed by WB for β-actin to ensure that equal amounts of proteins were used for each cell line. IP and control boxes are cut from the same gel at different exposure times, and properly aligned according to the molecular weight standard loaded onto the gel. Full length blots with molecular markers are shown in Supplementary information (Supplemental Fig. S12). Representative gels out of two independent experiments, demonstrating co-IP of E6AP with p53 in both cell lines and higher p53 poly-ubiquitination in SCCF3 are shown. E6AP was immunoprecipitated by incubation of SCCF2 (**b**) and SCCF3 **(c)** cell lysates with the specific antibody and the presence of p53 in the immune-complex as well as in input and supernatants (SN) revealed by WB using enhanced ECL reagents. The specificity of the co-IP was checked by parallel incubation with beads alone (Beads) and non-specific antibodies (IgG). The same membrane was blotted for E6AP to confirm the IP. Input samples were loaded onto the same gel to check for the right molecular weight of the co-IP bands and calculate the percentage of p53 bound to E6AP. IP and input boxes are cut from the same gel at the same exposure time and properly aligned according to the molecular weight standard loaded onto the gel. Representative gels demonstrating co-IP of p53 with E6AP are shown.

Once we demonstrated that E6AP is bound to p53 in presence of FcaPV-2 E6, we checked whether this may also occur in SCCF2 and SCCF3 cells by investigating the possible co-IP of E6AP with p53 as above. The p53 protein was immunoprecipitated at higher amount in SCCF2 cell line (Fig. 6a). Although at lower levels and longer exposure times, p53 was successfully detected by IP also in SCCF3 cells (Fig. 6a), due to sample enrichment with respect to simple WB. E6AP was co-immunoprecipitated with p53 in both cell lines, indicating that they were physically bound also in these cell models (Fig. 6a). Interestingly, when incubating the membrane with anti-ubiquitin antibody, higher poly-ubiquitinated p53 levels were detected in SCCF3 IP lane compared to SCCF2 (Fig. 6a). To possibly hypothesize that this might be due to higher levels of binding to E6AP, the reverse co-IP was performed and the percentage of p53 bound to E6AP calculated. The results confirmed the specificity of the co-IP and densitometric analysis of input and co-IP bands revealed that increased amount of p53 was bound to E6AP in SCCF3 with respect to SCCF2 (4% vs 0.2%, respectively) (Fig. 6b,c). It should be noted that, in this case, enhanced ECL reagent was necessary to detect p53 co-immunoprecipitated with E6AP, as well as in input and IP supernatant. These data suggested that enhanced E6AP mediated poly-ubiquitination and degradation of p53 would contribute to the lower to almost undetectable p53 protein levels in SCCF3 with respect to SCCF2. Moreover, E6AP siRNA yielded accumulation of p53 with respect to scramble-treated and untreated SCCF3 cells (Supplemental Fig. S8), suggesting that p53 protein stability was actually E6AP dependent in this cell line.

### FcaPV-2 DNA and mRNA detection in feline SCC samples

Once we obtained consistent results in two different *in vitro* models, we aimed at extending our analysis *in vivo* in FFPE tumour samples. Qualitative Real-time PCR was performed for detection of viral DNA (data not shown). Five (T1-T5) out of seventeen SCC samples (29%) were found to harbour FcaPV-2 DNA. Feline β-globin was successfully amplified from all the samples.

Only FcaPV-2 DNA positive samples were considered for RNA analysis. Of these, 5/5 were found to express mRNA encoding for viral oncogenes by RT-PCR (Supplemental Fig. S9).

Results of viral investigations on tumour samples are summarized in Table 1.

### Analysis of p53 and E6AP localization in feline SCC samples

**Figure 7:**
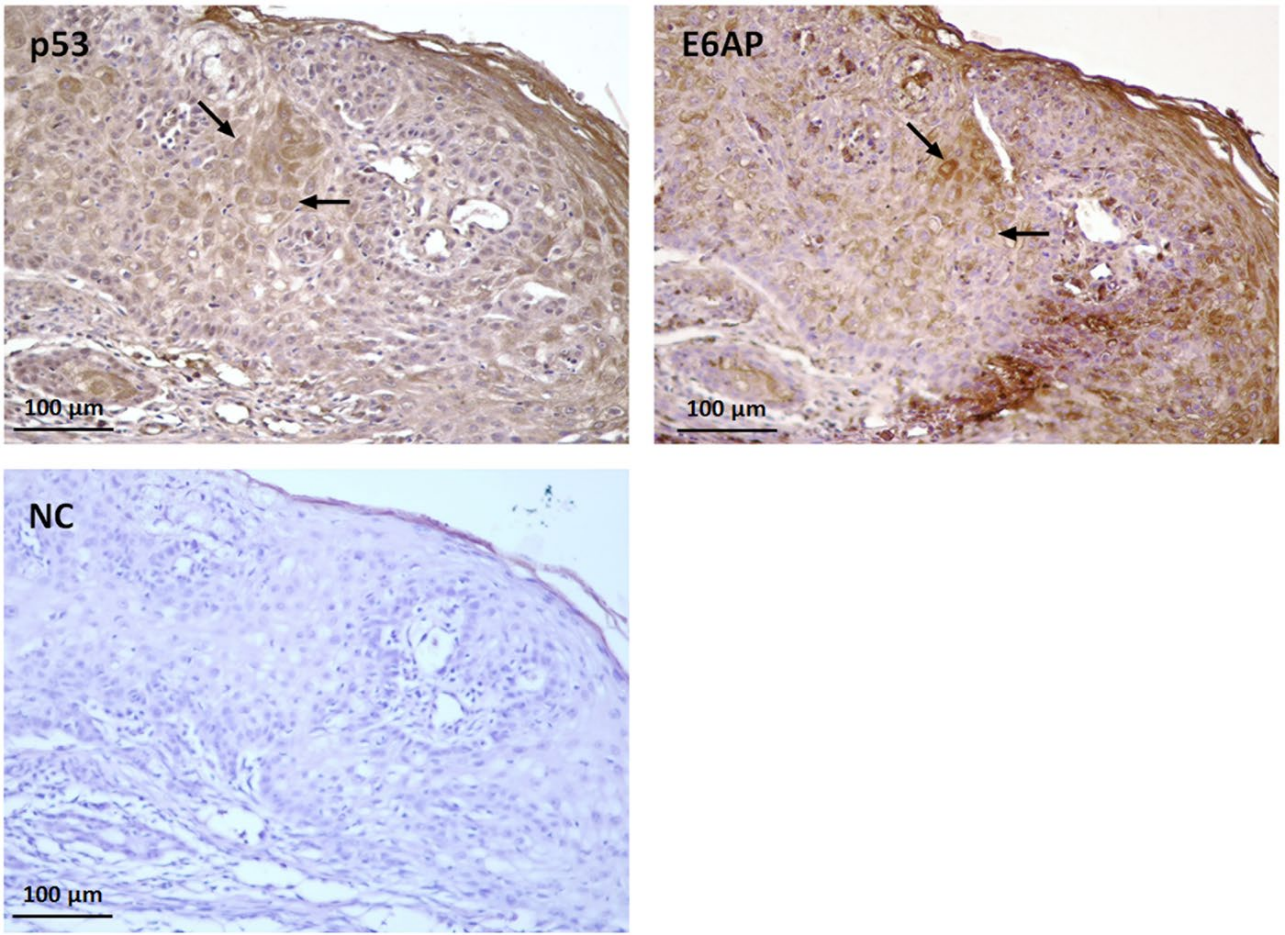
Expression and localization of p53 and E6AP in feline SCC. Serial sections of formalin-fixed paraffin embedded feline SCC were analysed by immunohistochemistry for p53 and E6AP using streptavidin-avidin method. Bound antibodies were visualized with 3,3′-diaminobenzidine tetrahydrochloride, nuclei were counterstained with Mayer’s haematoxylin. Representative micrographs showing cytoplasmic co-expression of the two proteins (black arrows) in scattered squamous cells within the SCC (T3) are illustrated. Negative control (NC) with primary antibody omitted is also shown.

Three (T1, T3, T5) out of 5 mRNA positive samples (60%) were available for IHC analysis on serial sections. The slides were stained with anti-p53 and anti-E6AP antibodies in order to assess their possible co-expression in the same cells within the SCC. In all the analysed samples, most of the tumour section was negative for p53 expression, however several cells in few neoplastic nests showed a weak to moderate cytoplasmic staining. Nuclear expression was observed in rare cells. Cytoplasmic expression, mostly with a perinuclear pattern, was recorded for E6AP in all the samples. Interestingly, scattered squamous tumour cells showed cytoplasmic co-expression of p53 and E6AP, resembling the results obtained *in vitro*. Representative IHC co-expression experiments are shown in Fig. 7 and Supplemental Fig. S10. As expected, feline mammary adenocarcinoma run as positive control showed p53 staining. Negative controls with primary antibodies omitted did not stain.

## Discussion

The E6 from mucosal HR HPVs promotes the assembly of a ternary complex with E6AP and p53, causing enhanced ubiquitination and proteasomal degradation of the latter^2^. Expression of FcaPV-2 E6 in feline cells promotes the reduction of p53 protein levels, suggesting similar biological properties to mucosal HPVs^5^. Hence, we aimed at unravelling the molecular mechanism of E6-mediated p53 down-regulation and whether it involved E6AP, in our previously-established *in vitro* model of viral pathogenesis. It is worthwhile to point out that the formation of HR HPVs E6/E6AP heterodimer is unavoidably required for their interaction with p53 and neither E6 nor E6AP are separately able to recruit p53^3,24,27,28^. The binding of p53 with FcaPV-2 E6 had been already demonstrated in CRFK cells^5^, then the complementary interactions were verified. The co-IP of E6AP with FcaPV-2 E6 confirmed the ability of the viral oncoprotein to recruit the ubiquitin ligase and this, along with E6AP co-IP with p53 exclusively in CRFKE6, suggests the occurrence of the triple binding. In the HPV pathogenic model, the ternary complex and the subsequent p53 degradation are believed to occur in the perinuclear region^29^; consistently, p53 and E6AP as well as FcaPV-2 E6 and p53 proteins showed a marked cytoplasmic co-localization in CRFKE6, mainly with a perinuclear like pattern, therefore a similar molecular scenario is conceivable in our feline model. Conversely, nuclear localization of p53 in mock-transfected cells is consistent with its normal function as transcription factor in tumour suppressor activities^30^. Furthermore, a poly-ubiquitinated form of p53 with high molecular mass was detected at higher levels in cells expressing FcaPV-2 E6. A similar ubiquitination pattern occurs in presence of HR HPVs E6, which promotes direct conjugation of high molecular weight poly-ubiquitin chains rather than the typical ladder of multiple mono-ubiquitinated forms of p53, thus resulting in its faster degradation^23–25^; moreover, FcaPV-2 E6 knock-down caused a shift in p53 ubiquitination pattern and degree, further suggesting that the viral oncoprotein was responsible for this. Consistently, half-life experiments revealed accelerated p53 degradation in CRFKE6, where higher p53 accumulation upon proteasome inhibition confirmed that this phenomenon was proteasomal dependent. Importantly, E6AP gene knockdown yielded p53 rescue in CRFKE6 but not CRFKpCEFL, demonstrating that p53 protein stability was dependent from E6AP in presence of FcaPV-2 E6.

Considered overall, these data suggest that FcaPV-2 E6 triggers the assembly of the canonical ternary complex with E6AP and p53, leading to its enhanced ubiquitination and proteasomal degradation, similarly to HR HPVs E6. Moreover, lower E6AP levels in cells expressing FcaPV-2 E6 might indicate an additional similarity with mucosal HPVs, considering that HPV-16 E6 causes E6AP degradation as well^31^; however, this topic will be deepened in future studies.

There is increasing evidence that HR HPVs play a key role also in the development of a distinct sub-category of oral SCC, accounting for the 25% of HNSCC^4^. Feline oral SCC is considered a spontaneous animal model of human HNSCC, since similar histopathological and biological features (for instance genetic alterations and protein expression profiles)^6,7^ exist between HNSCC of the two species. Additionally, the two oncological entities share several risk factors, such as exposure to environmental chemicals^6,7^. The presence of FcaPVs in feline oral SCC and pre-neoplastic lesions has been reported but is infrequent to date, thus the possible role in the aetiology of this feline cancer is still uncertain^5,8–12^. Therefore, we extended our molecular studies in living cells from naturally occurring feline cancer, in order to obtain a first descriptive outlook on their molecular scenario. Both SCCF2 and SCCF3 cells harboured FcaPV-2 DNA and expressed E6 oncogene. Notably, SCCF3 cell line showed higher E6 gene expression along with lower, almost undetectable p53 protein levels with respect to SCCF2. E6AP and p53 were physically bound in both cell lines, however the amount of p53 bound to E6AP as well as p53 poly-ubiquitination degree, were higher in SCCF3, suggesting that the activity of the E6-driven p53 degradation machinery was proportional to E6 expression levels. And yet, siRNA experiments confirmed that p53 protein stability was strongly dependent on E6AP in SCCF3, in agreement with what previously reported in HR HPV-positive cell lines^32^. A lower efficiency of p53 binding to E6AP in SCCF2 is also consistent with the IF data showing their cytoplasmic/perinuclear co-localization only in a sub-set of cells, likely those infected by FcaPV-2. Nevertheless, higher E6 gene expression might explain the prevalent perinuclear localization of E6AP in SCCF3, in accordance with what stated above for CRFKE6^7^. We have previously shown that additional FcaPV-2 oncogenes may impact on p53 amount through down-regulation of its mRNA levels in feline SCC and cultured cells^5^. Therefore, it is plausible that the lower p53 transcript in SCCF3 compared to SCCF2 may be due to higher expression levels of these additional oncogenes along with E6, considering that PVs oncoproteins are translated from a unique polycistronic mRNA^33^. This might confirm that FcaPV-2 could have evolved different strategies to redundantly impair p53 also in feline oral SCC. Therefore, the results obtained in cell lines derived from spontaneous tumours suggest that FcaPV-2 might play a role in the development of a sub-set of feline oral SCC and, remarkably, they are consistent with those ensued in our previously established cell model, strengthening the reliability of the pathogenic pathway proposed in this study. However, further mechanistic studies are warranted to definitely validate the molecular mechanism in SCCF cell lines.

Of note, the data obtained in FcaPV-2 positive SCC samples were consistent with those observed in cultured cells: p53 expression was lost in most of each tumour section, except in rare cells where it was co-expressed with E6AP with a cytoplasmic/perinuclear pattern, suggesting a similar molecular mechanism occurring *in vivo*. In previous studies, ∼60% of the analysed feline skin SCCs did not stain for p53^34,35^, consistently, at least in part, with our data; in the remaining ∼40% of positive samples, the staining was restricted to the nuclei, however p53 expression and staining pattern have never been correlated with FcaPV-2 oncogenes expression. Thus, the possibility that those samples did not harbour transcriptionally active FcaPV-2 might explain the apparent discrepancy with our results. Moreover, these works have not specifically analysed feline oral SCC for the presence of FcaPV-2 and association with p53 differential expression and/or localization. Therefore, additional studies on larger sets of clinical cases would be required to further confirm the association of p53, E6AP, and FcaPV-2 E6 *in vivo* and possibly assume feline oral SCC as a model of HPV-driven HNSCC.

In conclusion, this study suggests that FcaPV-2 shares the mechanism of p53 degradation with mucosal HR HPVs and that it might be able to infect oral cavity and contribute to the development of SCC in this anatomical site. It is worthwhile noting that these conclusions were drawn by integrating results from artificial and spontaneous *in vitro* experimental models along with tumour samples. Thus, in a future perspective, this work paves the way for additional integrated functional studies to better characterize the biology and oncogenic potential of FcaPV-2.

## Electronic supplementary material

Supplementary information

## Data Availability

The datasets generated and/or analysed in this study (and its Supplementary Information files) are available from the corresponding author on reasonable request.
