# Supplementary material for: Felis catus papillomavirus type-2 E6 binds to E6AP, promotes E6AP/p53 binding and enhances p53 proteasomal degradation
Source: Sci Rep. 2018 Dec 3;8:17529. doi: 10.1038/s41598-018-35723-7 (PMC6277439; doi:10.1038/s41598-018-35723-7)
Supplement: Supplementary file 1 — Supplementary information [file 41598_2018_35723_MOESM1_ESM.pdf]

Felis catus papillomavirus type-2 E6 binds to E6AP, promotes E6AP/p53 binding and enhances p53 proteasomal degradation

Gennaro Altamura[1], Karen Power[1], Manuela Martano[1], Barbara degli Uberti[2], Giorgio Galiero[2], Giovanna De Luca[2], Paola Maiolino[1] and Giuseppe Borzacchiello[1]*

[1] Department of Veterinary medicine and Animal production – University of Naples Federico II – Via Veterinaria 1, Naples, 80137, Italy
[2] Istituto Zooprofilattico Sperimentale del Mezzogiorno, Via Salute 2, Portici, Naples, 80055, Italy

*Corresponding author:
Prof. Giuseppe Borzacchiello
Department of Veterinary medicine and Animal production
University of Naples Federico II
Via Veterinaria 1, Naples, 80137, Italy.
Tel: +390812536467
e-mail: borzacch@unina.it.

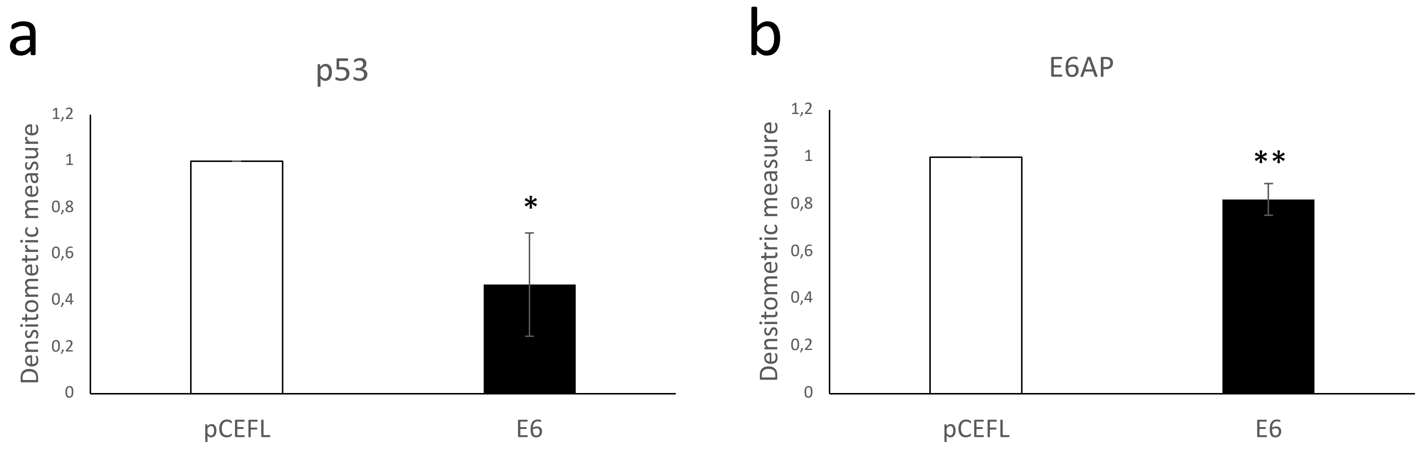

Supplemental Figure S1.
Mean densitometric values +/- standard deviations from three independent Western blotting for p53 **(a)** (*t-test*, P=0.0144) and E6AP **(b)** (*t-test,* P=0.0099) in CRFKpCEFL and CRFKE6.

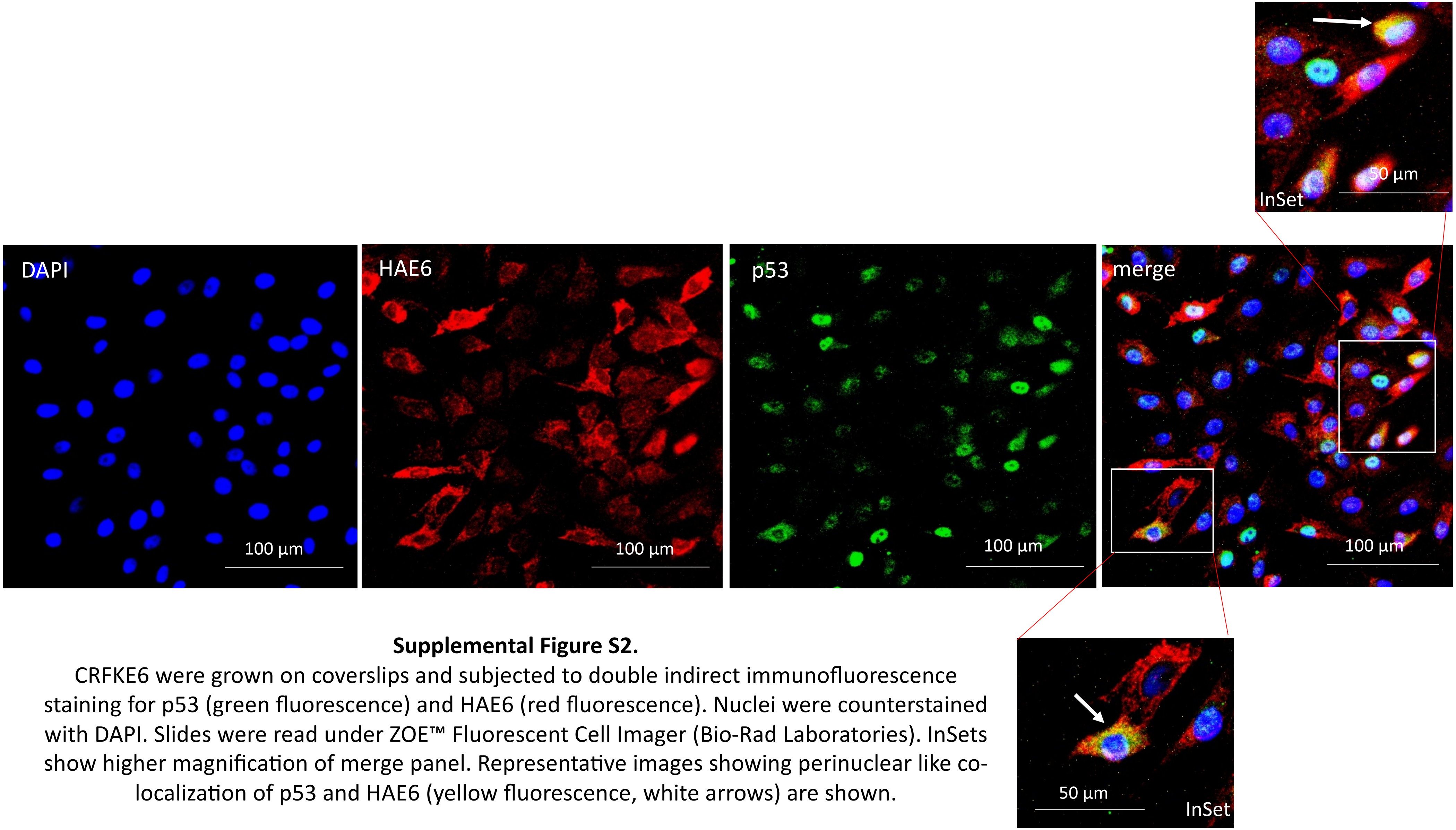

Supplemental Figure S2.

CRFKE6 were grown on coverslips and subjected to double indirect immunofluorescence staining for p53 (green fluorescence) and HAE6 (red fluorescence). Nuclei were counterstained with DAPI. Slides were read under ZOE™ Fluorescent Cell Imager (Bio-Rad Laboratories). InSets show higher magnification of merge panel. Representative images showing perinuclear like co-localization of p53 and HAE6 (yellow fluorescence, white arrows) are shown.

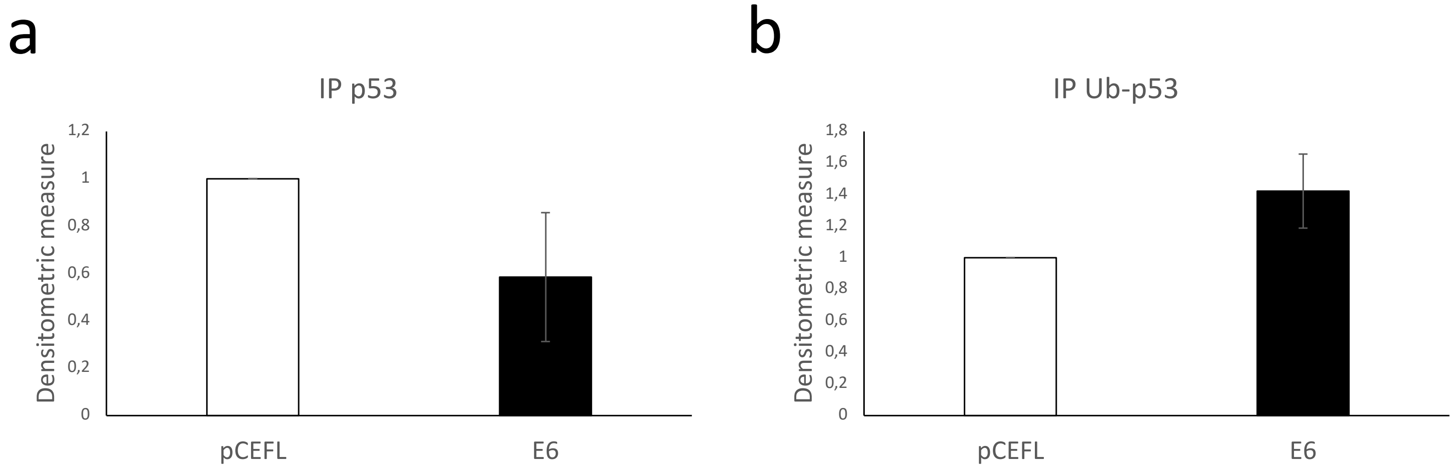

Supplemental Figure S3.
Mean densitometric values +/- standard deviations from two independent experiments, showing lower levels of immunoprecipitated (IP) p53 **(a)** and higher levels of IP Ubiquitinated p53 (Ub-p53) **(b)** in CRFKE6 compared to CRFKpCEFL.

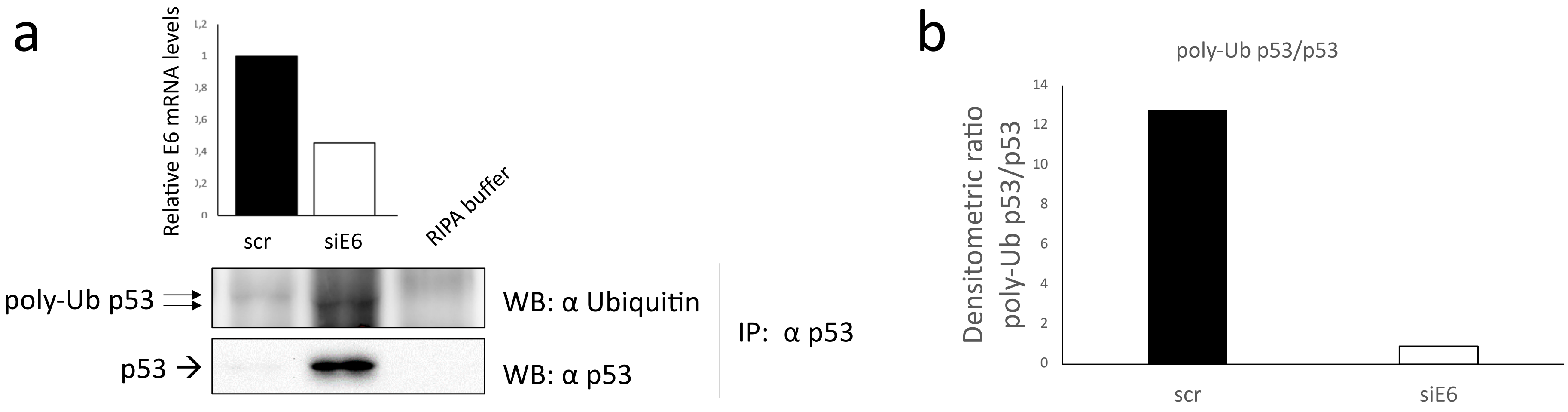

Supplemental Figure S4.

(a) CRFKE6 were subjected to E6 siRNA (siE6) and scramble (scr) treatment and analysed by Real-time PCR. From the same samples, the same amounts of cell lysates (and RIPA buffer as control) were immunoprecipitated by incubation with anti-p53 antibody, and the presence of ubiquitin in the immunocomplex revealed by WB. The membrane was blotted for p53 to confirm the IP. The results demonstrate the decrease in E6 gene expression along with a change in p53 ubiquitination pattern and p53 accumulation in cells subjected to siE6. **(b)** Densitometric analysis of poly-ubiquitination of p53 in scr *vs* siE6 treated cells, the results are shown as poly-ub p53/p53 densitometric ratio.

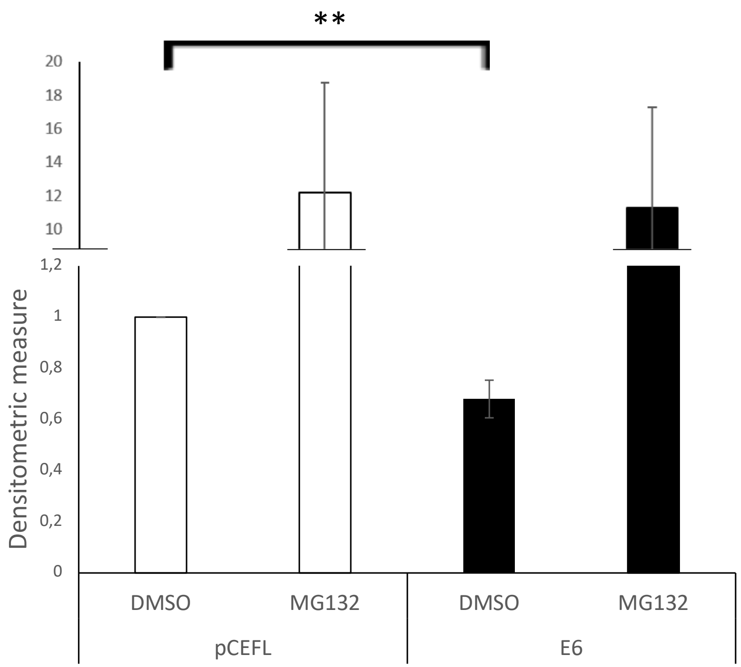

Supplemental Figure S5.
Densitometric analysis of the experiment shown in Figure 3c and 3d. Data are presented as mean +/- standard deviations of raw densitometric values from four independent experiments (*t-test,* P=0.0001).

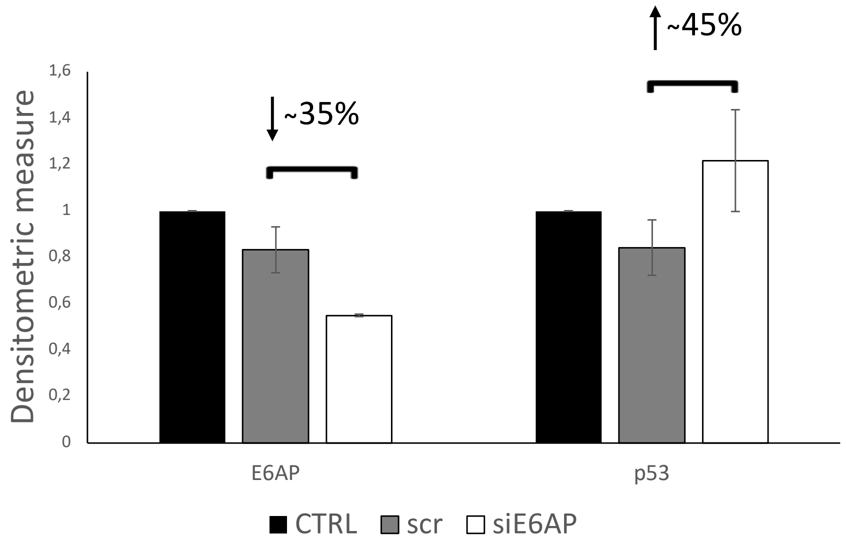

Supplemental Figure S6.

Mean densitometric values +/- standard deviations from two independent experiments of E6AP siRNA (siE6AP) in CRFKE6. The data show increased levels of p53 (~45%) along with a decrease of E6AP levels (~35%) in siE6AP compared to scramble-treated (scr) cells.

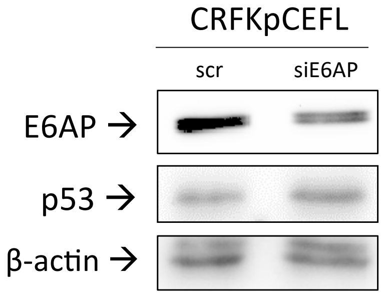

Supplemental Figure S7.
CRFKpCEFL were transiently transfected with 30 nM siRNA directed against E6AP (siE6AP) or with scramble RNA (scr) and analysed by WB for p53 and E6AP. The blot was stripped and reprobed for β-actin to ensure that equal amounts of protein were loaded in each lane.

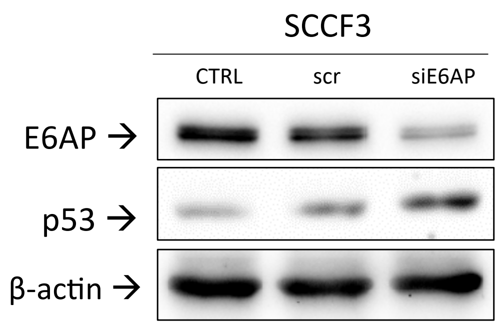

Supplemental Figure S8.

SCCF3 cells were transfected with 30 nM scramble RNA (scr) or E6AP siRNA (siE6AP) oligonucleotides and analysed by WB for p53 and E6AP. The gel shows accumulation of p53 upon E6AP gene silencing compared to scramble-treated (scr) and untreated cells (CTRL). The membrane was stripped and reprobed with anti-β-actin antibody to ensure equal protein loading in each lane.

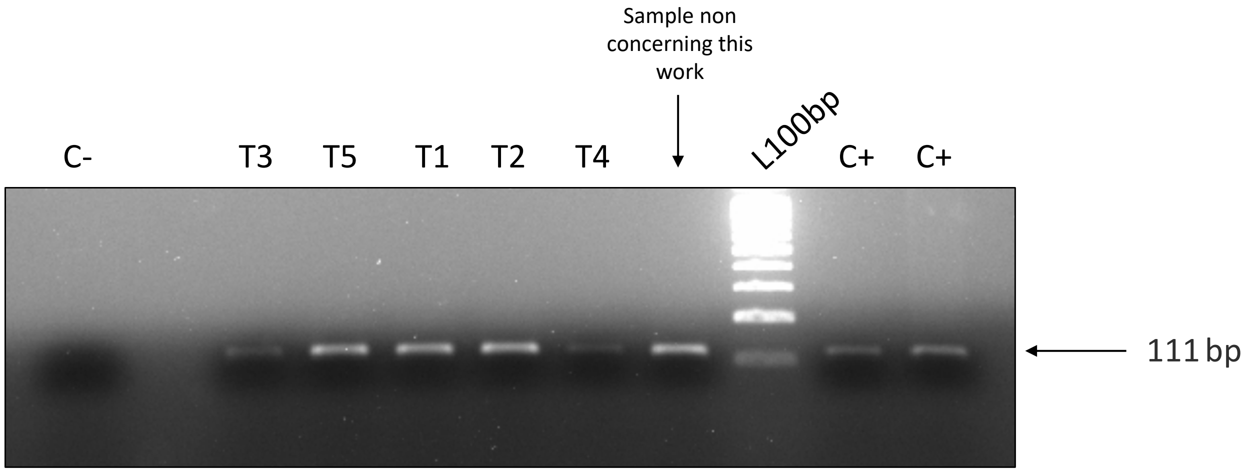

Supplemental Figure S9.

RT-PCR gel demonstrating detection of a FcaPV-2 mRNA in feline SCC samples. A fragment of the expected size (111bp) was successfully amplified from samples T1-T5 and FcaPV-2 genome used as positive control (C+). The size of the amplicon is indicated on the right of the panel (C−: negative control with no template; L100bp: 100 base pairs DNA ladder, the first band from the bottom is 100 bp).

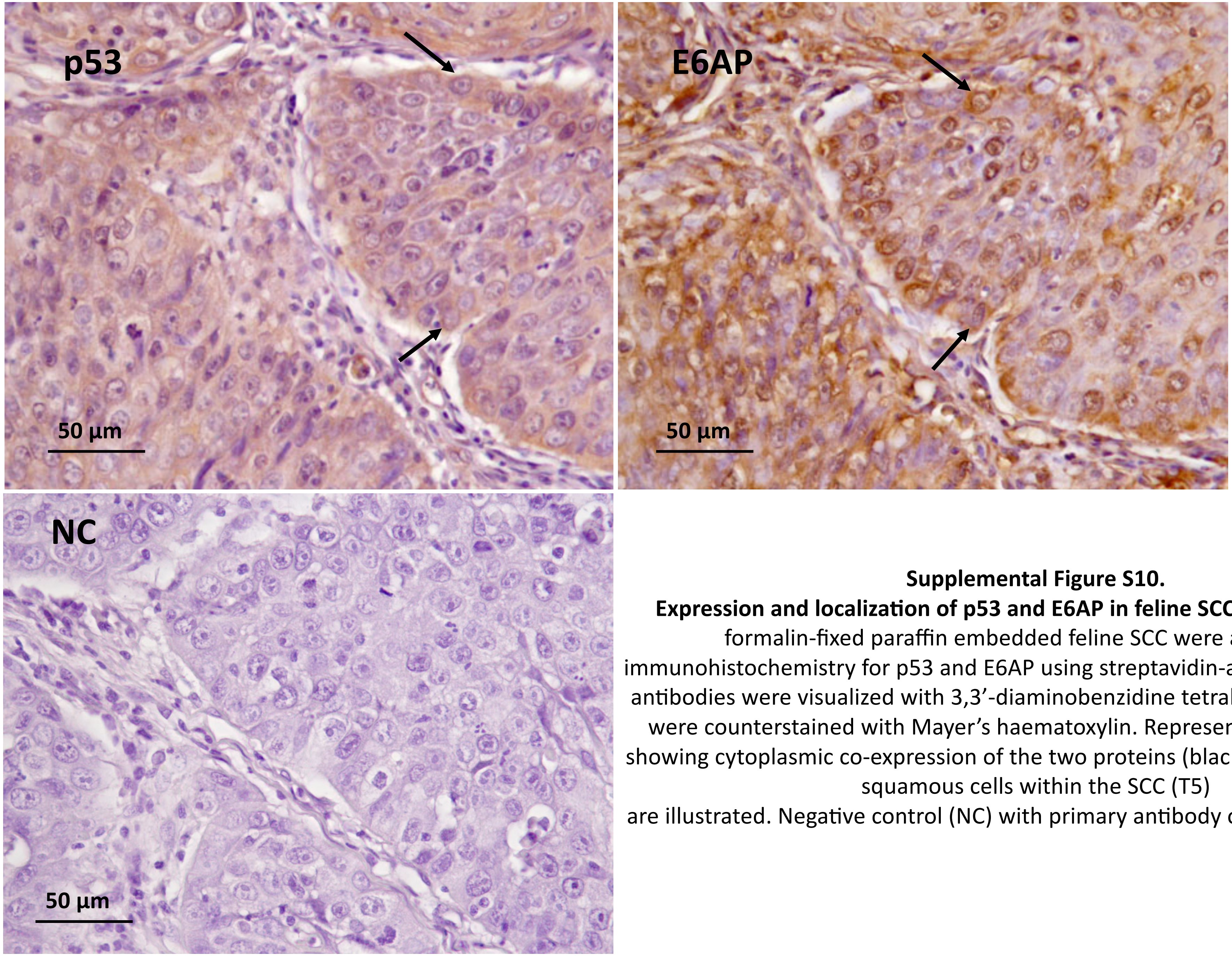

Supplemental Figure S10.

Expression and localization of p53 and E6AP in feline SCC. Serial sections of formalin-fixed paraffin embedded feline SCC were analysed by immunohistochemistry for p53 and E6AP using streptavidin-avidin method. Bound antibodies were visualized with 3,3'-diaminobenzidine tetrahydrochloride, nuclei were counterstained with Mayer's haematoxylin. Representative micrographs showing cytoplasmic co-expression of the two proteins (black arrows) in scattered squamous cells within the SCC (T5) are illustrated. Negative control (NC) with primary antibody omitted is also shown

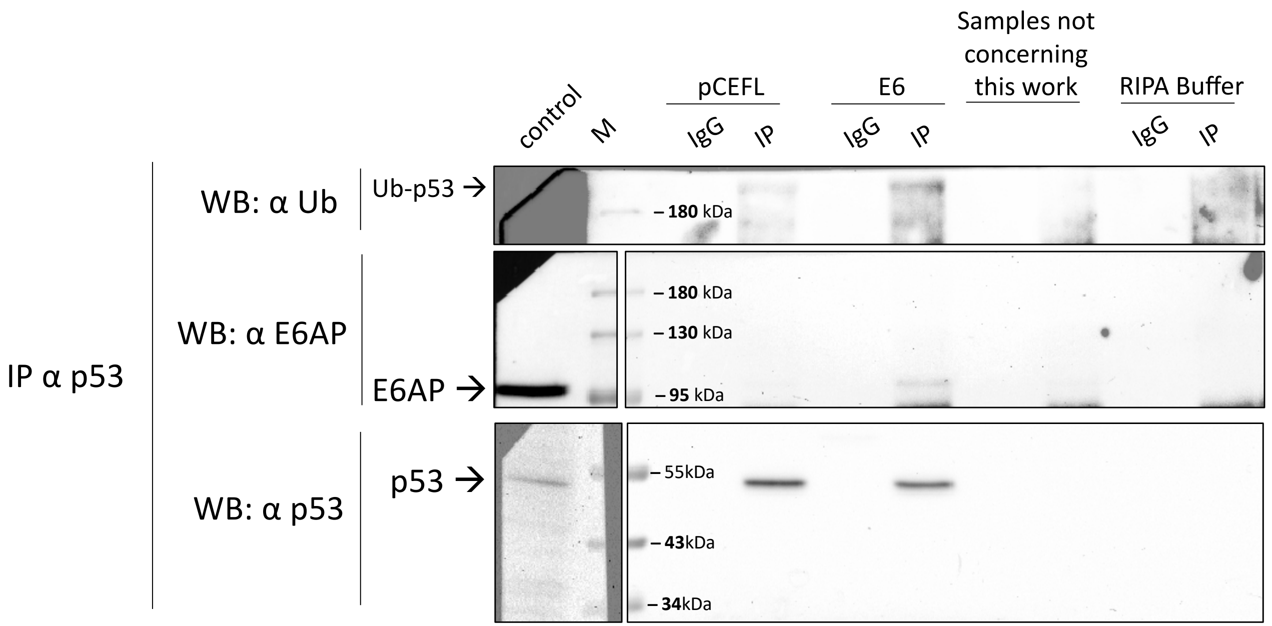

Supplemental Figure S11.

Full length blots of co-immunoprecipitation experiments from Figure 2b demonstrating physical interaction of E6AP with p53 and higher Ub-p53 levels in CRFKE6. Molecular markers (M) are shown, molecular weights are indicated on the right of each M band.

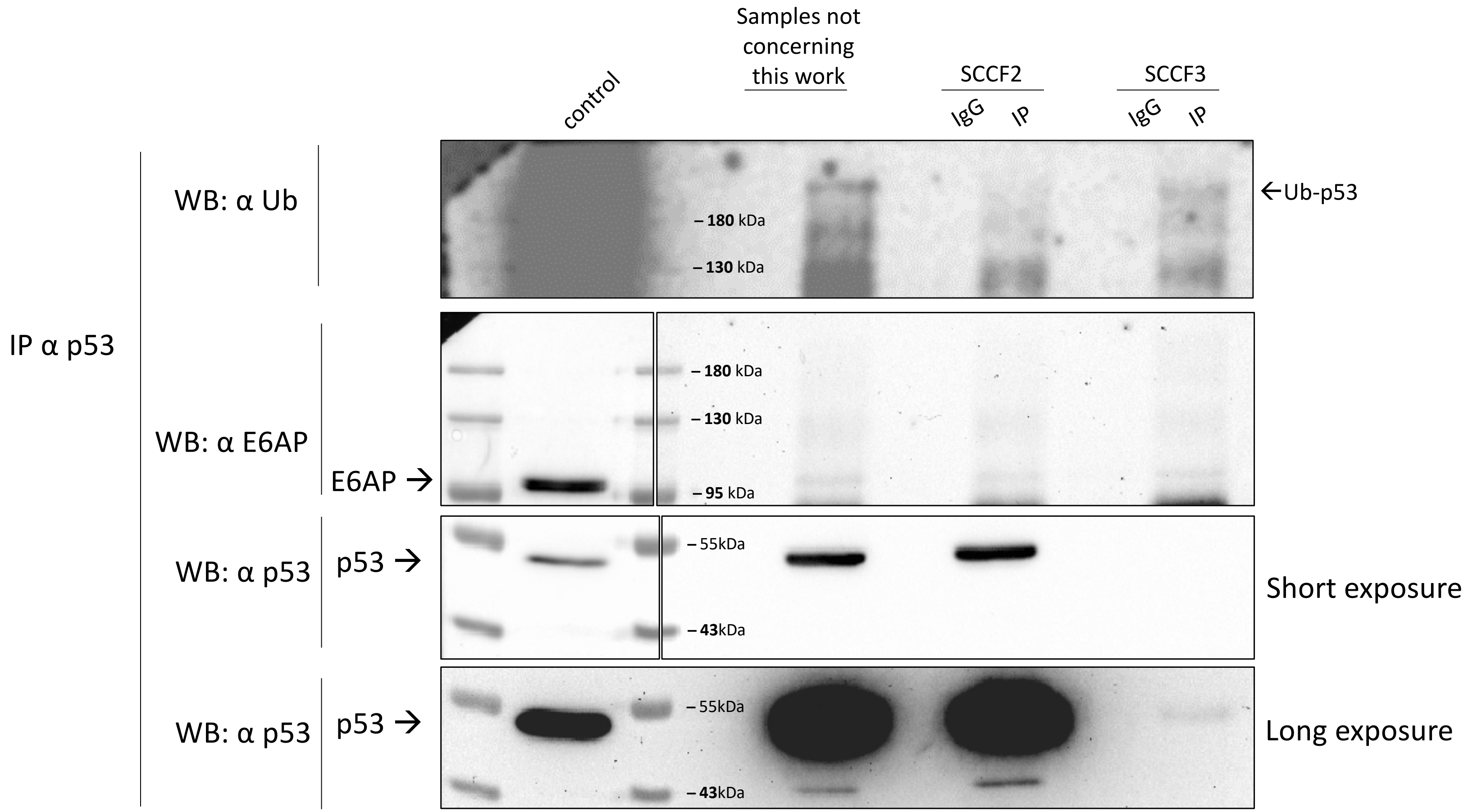

Supplemental Figure S12.

Full length blots of co-immunoprecipitation experiments from Figure 6a demonstrating physical interaction of E6AP with p53 in SCCF2 and SCCF3 and higher Ub-p53 levels in SCCF3. Molecular markers (M) are shown, molecular weights are indicated on the right of each M band.

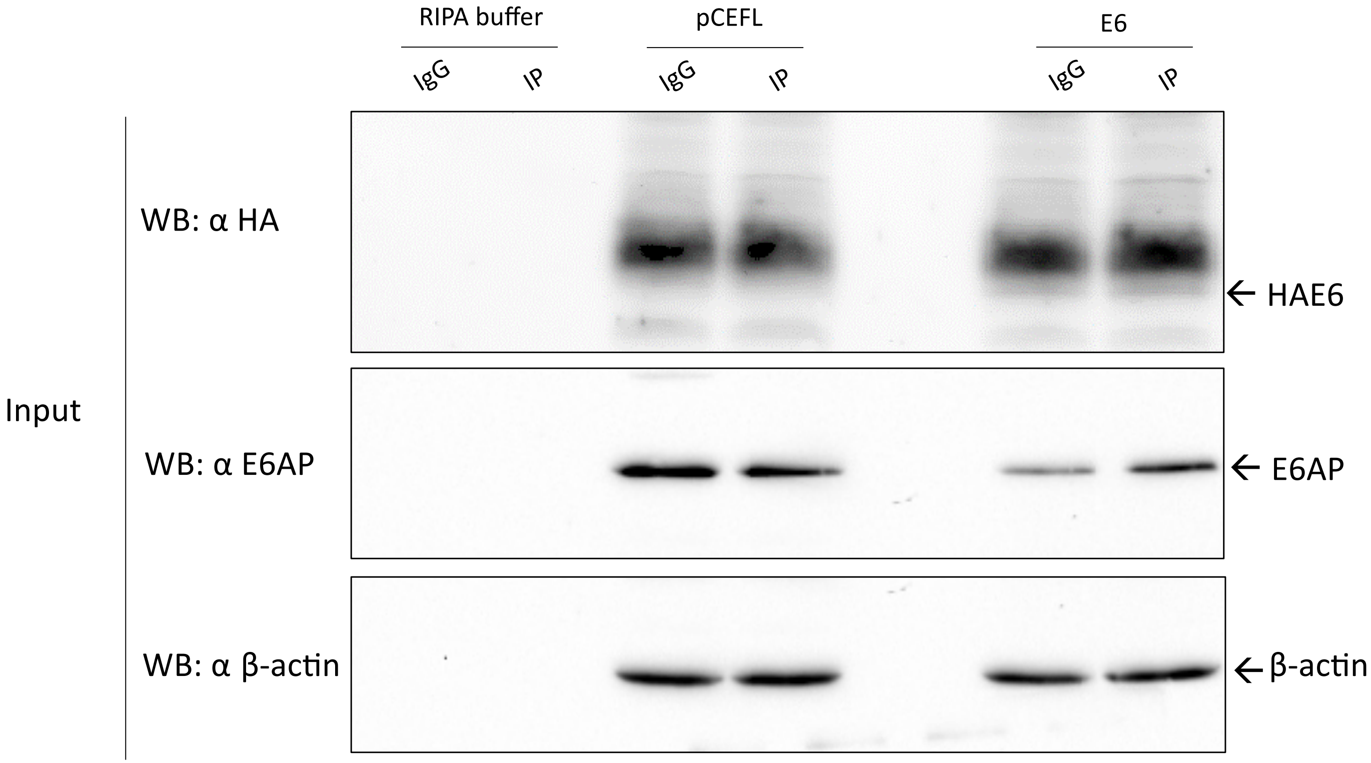

Supplemental Figure S13.
Full length blots of input samples from co-immunoprecipitation experiments shown in Figure 2a.
